# Geospatial analysis enables combined poultry–fish farm monitoring in the fragile state of Myanmar

**DOI:** 10.1038/s43016-025-01192-1

**Published:** 2025-07-23

**Authors:** Ben Belton, Peixun Fang, Shuo Liu, Kaifeng Zhang, Xiaobo Zhang

**Affiliations:** 1 International Food Policy Research Institute, Dhaka, Bangladesh; 2 Michigan State University, East Lansing, MI USA; 3 International Food Policy Research Institute, Washington, DC USA; 4 Peking University, Beijing, China; 5 Columbia University, New York, USA

**Keywords:** Economics, Development studies

## Abstract

Food security is challenging to measure in fragile contexts. Here we combine data from previous field surveys with remotely sensed images and apply deep-learning techniques to estimate changes in the number and area of chicken houses on integrated chicken–fish farms and the supply of chicken meat and eggs from 2010 to 2023 in Yangon region, Myanmar. Yangon’s poultry sector grew ~10% annually from 2010 to 2020 but contracted ~8% annually from 2020 to 2023.

## Abstract (web summary)

Accurate data collection remains challenging in fragile contexts, which are prone to food insecurity. Remote sensing, survey data and deep learning can generate poultry–fish sector production estimates and contribute towards long-term food security monitoring.

## Main

About a quarter of the world’s population and three-quarters of extremely poor people live in a fragile state, which is defined as the combination of exposure to risk and insufficient coping capacities of the state and/or communities to manage, absorb or mitigate those risks^1^. Conflict and state fragility are major drivers of food insecurity^2^. Food insecurity monitoring in conflict-affected areas is necessary to track the impacts of shocks and support the design of effective interventions^2^. Yet because safe and reliable data collection in fragile and violent environments is challenging, there is little research on agricultural production in these areas^3^.

Telephone surveys were widely used to track food insecurity during the COVID-19 crisis^4^, but suffer from limitations including sampling biases, high attrition rates and human resource constraints^5^. An alternative approach to tracking food system performance is to apply machine-learning techniques to data obtained from remote sensing and conventional household surveys^6–10^. We extend this general approach to estimate changes in poultry production in Yangon region, Myanmar, during a period of political instability and conflict.

Myanmar experienced a decade of political reform and economic growth from 2010 to 2019, during which some agricultural subsectors, including feed-lot poultry farming, expanded rapidly^11,12^. The growth trajectory of Myanmar’s economy and poultry industry was halted by the COVID-19 pandemic in 2020^12^. In early 2021, Myanmar’s military seized control of the country in a bloody coup, precipitating widespread violence and rapid economic collapse^3^. By 2022, 15.2 million people faced acute food insecurity^13^, and by June 2023, 61% of Myanmar’s population were income poor^14^.

In Myanmar, chickens are often raised in poultry houses built over fish ponds. This design allows manure and uneaten feed to fall into ponds below, where they become a source of nutrients for fish cultivation^12^. We trained an algorithm to detect chicken houses on integrated chicken–fish farms using satellite images and reference information from previous field surveys. This approach enabled us to estimate the number of poultry houses on integrated chicken–fish farms in Yangon region over time, and their size and the quantity of birds and eggs produced.

The model performs very well. It identified 1,750 integrated chicken–fish houses in Yangon region in 2017, close to the 1,508 farms reported by a livestock census in the same year^15^. Table 1 presents the number and average size of chicken houses on integrated chicken–fish farms detected from Google Earth^16^ satellite images between 2010 and 2023. The number of chicken houses increased by 10.7% per year, from less than 1,000 in 2010 to more than 2,500 in 2020, an overall increase of 160%. The total area of chicken houses grew faster still, by 13% per year (222% overall), as the average size of individual houses also increased. This increase was linked to a change in construction materials, with larger zinc-roofed houses increasingly favoured over smaller houses with thatched roofs (Supplementary Table 4).

**Table 1:** Number and average size of chicken houses on integrated chicken–fish farms in Yangon region

Year	Total number of houses	Total area of houses (m^2^)	Mean area per house (m^2^)
2010	976	401,741	411
2011	1,043	439,268	421
2012	1,172	504,298	430
2013	1,230	531,144	432
2014	1,412	676,835	479
2015	1,471	705,982	480
2016	1,573	760,597	483
2017	1,750	877,472	501
2018	2,340	1,174,742	502
2019	2,487	1,277,860	514
2020	2,542	1,292,753	509
2021	2,141	1,102,998	515
2023	1,954	1,066,746	546
Overall change 2010–2020 (%)	160.4	221.8	23.6
Overall change 2020–2023 (%)	−23.1	−17.5	7.4
Annual change 2010–2020 (%)	10.7	13.3	2.4
Annual change 2020–2023 (%)	−7.9	−5.6	2.5

Source: Google Earth satellite images.

**Fig. 1:**
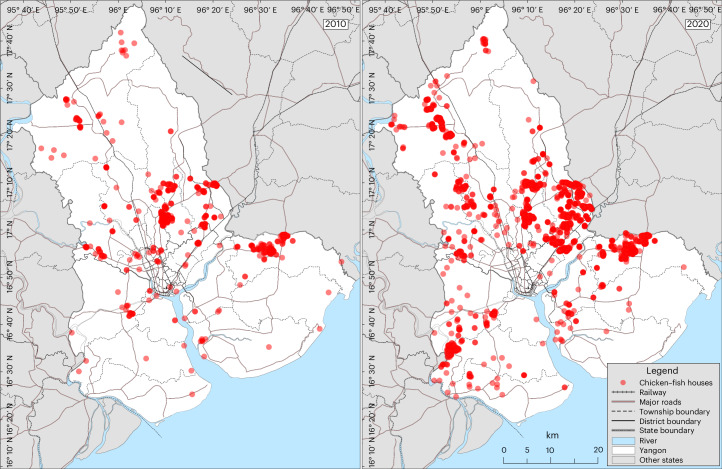
The spatial distribution of integrated chicken–fish farms in Yangon region. The spatial distribution in 2010 (left) and 2020 (right). Red dots represent individual chicken houses. Source: authors’ compilation on background maps from Myanmar Information Management Unit^20^. Source data

Figure 1 depicts the spatial distribution of integrated chicken–fish farms in Yangon region in 2010 and 2020. In 2010, only two major clusters of farms existed. By 2020, several new clusters had emerged around nearby towns along main roads to the city.

From 2020 to 2023 the number of integrated chicken–fish houses fell sharply, by 7.9% per year (23.1% overall), probably due to the economic effects of the COVID-19 pandemic and subsequent political shocks^12^.

We combined information from satellite images with data from an earlier field survey to estimate changes in the supply of chicken meat and eggs from integrated chicken–fish houses in Yangon region from 2020 to 2023. We estimate that the standing population (number of birds housed at any given point in time) on integrated chicken–fish farms in Yangon region fell by approximately 1 million broilers and 0.55 million layers between 2020 and 2023, equivalent to 10,100 t less broiler meat and 5,700 t less eggs per year in 2023 than in 2020.

These losses resulted in an estimated decline in annual consumption per capita of chicken meat and eggs of 1.3 kg and 0.7 kg, respectively for inhabitants of Yangon, equivalent to an 11.6% fall in consumption of chicken meat and 16.4% fall in consumption of eggs from 2015 levels (Supplementary Table 5).

This paper has used the example of integrated chicken–fish production in Myanmar to demonstrate the potential of using remotely sensed data combined with information from existing field surveys to track the performance of agriculture in fragile states. Our model predictions match well with data from field surveys conducted prior to 2020 and provide a detailed picture of the direction and scale of sectoral growth. The model is particularly useful in providing information on trends since 2020, a turbulent period for Myanmar, during which in-person data collection has been impossible. Our estimates are consistent with a telephone survey conducted in 2020, which confirmed that many poultry farms shut down in that year^12^.

The method presented here offers two major advantages over the telephone survey approach. First, remote sensing allows for continuous tracking of the sector across a wide geographical area, providing a more complete picture of its evolution. By comparison, the telephone survey was limited to a relatively small convenience sample that underwent attrition and ended in late 2020. Second, remote sensing is a more cost-effective means of data collection because of lower human resource requirements.

Importantly, both approaches relied on earlier in-person field surveys. The remote sensing-based approach uses survey data to estimate changes in the supply of chicken and eggs, and the telephone survey sample frame and questionnaire were based on an earlier in-person survey. This observation underlines the importance of conducting regular agricultural monitoring surveys during ‘normal’ times, both for the information they provide, and because they can be a foundation for alternative data collection and monitoring approaches if circumstances change unexpectedly.

Although this Brief Communication has focused on integrated chicken–fish production, the approach may be adapted to estimate changes in food production and analyse implications for food security in other fragile, conflict-affected settings. For example, for tracking changes in crop production (for example, by estimating the area planted to specific crops), fisheries (for example, by estimating numbers of active fishing boats) and logistics (for example, by estimating numbers of granaries or cargo containers).

## Methods

To efficiently identify chicken houses within vast satellite images, we leveraged YOLOv4^17^, a convolutional neural network model that takes an RGB image as input, and outputs bounding boxes for target chicken houses with a confidence measure. We split the labelled data into training (80%), validation (10%) and testing (10%) sets. The model was trained on the training split, and hyperparameters were selected according to its performance on the validation set. The model’s accuracy of 99% provides strong evidence for its ability to accurately count chicken houses, even in the presence of some detection errors (Supplementary Table 1). This high accuracy suggests a near-equal balance between false negatives (missed houses) and false positives (incorrectly identified houses).

Our labelled data are derived from 2018 satellite imagery. We hypothesize that generalization to other years is straightforward due to the consistent resolution and visual style of the satellite images. To distinguish the roofing material of chicken houses, we adopted UNet^18^, a widely applied convolutional neural network model for image segmentation. The model’s validation intersection over union surpasses 80%, a remarkable achievement considering the limited labelled data (Supplementary Table 2). We also tried using the more recent SAM model. Our original UNet model performs much better. We believe that this is probably because SAM is trained using common images uploaded to the internet.

We used supporting data from two previous surveys in combination with analysis of satellite images to estimate changes in the supply of chicken and eggs from integrated farms in Yangon region. (1) The Yangon Peri-urban Livestock Survey, conducted in 2019, sampled commercial livestock farms within a 100-km radius of central Yangon^19^. The present study makes use of data from a subset of 286 integrated chicken–fish farms. (2) The Yangon Peri-Urban Poultry Farmer Survey^12^, conducted in 2020 by telephone over six rounds. This survey analysed the impacts of the COVID-19 pandemic on poultry production on farms close to Yangon. Results from our satellite-image-based algorithm for detecting chicken–fish houses in Yangon region were validated by comparison with data the government’s National Livestock Survey, conducted in 2018^15^. Our model identified 1,750 chicken–fish houses in Yangon region in 2017, which is close to the value of 1,508 reported by the National Livestock Survey.

To estimate change in chicken and egg supply in Yangon due to the closure of integrated chicken–fish farms between 2020 and 2023, we combined analysis of satellite images with data from previous conventional field surveys and secondary data sources, following the procedure reported in Supplementary Table 5. Finally, to evaluate chicken and egg consumption in Yangon region in 2023 against a historical baseline, we compared our estimate of average per-capita consumption with consumption in Yangon in 2015.

### Reporting summary

Further information on research design is available in the Nature Portfolio Reporting Summary linked to this article.

## Supplementary information

Supplementary Information Supplementary Figs. 1–3, Tables 1–5, Discussion and Methodology.

Reporting Summary

## Source data

Source Data Fig. 1 GPS locations of all identified integrated chicken–fish farms in the Yangon Region in 2010 and 2020.

## Data Availability

The Yangon Peri-urban Livestock Survey 2019 dataset is archived on Harvard Dataverse at: https://dataverse.harvard.edu/dataset.xhtml?persistentId=doi%3A10.7910%2FDVN%2FLKAQYF&version=DRAFT. The Yangon Peri-Urban Poultry Farmer Survey 2020 dataset is archived on Harvard Dataverse at: https://dataverse.harvard.edu/dataset.xhtml?persistentId=doi%3A10.7910%2FDVN%2FIY66P9&version=DRAFT. Source data are provided with this paper.

## Code availability

All STATA code used in the analysis of primary survey data and estimates of poultry and egg supply is available via GitHub at: https://github.com/Percyfang/Yangon-Chicken-Fish. All machine-learning code used in chicken house detection, segmentation and classification is available via GitHub at: https://github.com/PKUCER/YONGONCHICKENFISH.
