# Author Correction: Geospatial analysis enables combined poultry–fish farm monitoring in the fragile state of Myanmar

**DOI:** 10.1038/s43016-025-01249-1

**Published:** 2025-09-22

**Authors:** Ben Belton, Peixun Fang, Shuo Liu, Kaifeng Zhang, Xiaobo Zhang

**Affiliations:** 1 International Food Policy Research Institute, Dhaka, Bangladesh; 2 Michigan State University, East Lansing, MI USA; 3 International Food Policy Research Institute, Washington, DC USA; 4 Peking University, Beijing, China; 5 Columbia University, New York, USA

**Keywords:** Economics, Development studies

Correction to: *Nature Food* 10.1038/s43016-025-01192-1, published online 23 July 2025.

This paper was originally published under standard Springer Nature license (© The Author(s), under exclusive licence to Springer Nature Limited). It is now available as an open-access paper under a Creative Commons Attribution 4.0 International license, © The Author(s). The error has been corrected in the online version of the article.

