# Supplementary material for: Geospatial analysis enables combined poultry–fish farm monitoring in the fragile state of Myanmar
Source: Nat Food. 2025 Jul 23;6(7):664–7. doi: 10.1038/s43016-025-01192-1 (PMC12454148; doi:10.1038/s43016-025-01192-1)
Supplement: Supplementary file 1 — Supplementary Figs. 1–3, Tables 1–5, Discussion and Methodology. [file 43016_2025_1192_MOESM1_ESM.pdf]

nature food

Supplementary information https://doi.org/10.1038/s43016-025-01192-1

Geospatial analysis enables combined poultry–fish farm monitoring in the fragile state of Myanmar

In the format provided by the authors and unedited

Supplementary Information

Satellite images

Satellite images were collected from Google Earth Pro[1]. The database contains RGB images of different resolutions, taken at different times. We chose Yangon region (10,276.7 km^2) as the target area, as it contains the largest concentration of chicken farms in Myanmar[2]. We first accessed Google Earth data in 2021, downloading archived historical images for each year from 2010 to 2020 and 'live' images for 2021. Archived images of Yangon Region for 2022 have not yet been added to Google Earth. To access more recent data, we downloaded Google Earth live images in 2023, for a total 13 years of images (2010-2021, and 2023).

Our main targets were chicken houses integrated with (i.e. constructed above) fishponds. Chicken houses are long, thin, rectangular buildings sized approximately 550 m^2 per house. Fishponds are typically rectangular waterbodies surrounding chicken houses, averaging about 35,000 m^2 per pond. The color chicken house roofs is indicative of the roofing materials used (thatch and zinc), as shown in Supp. Fig. 1.

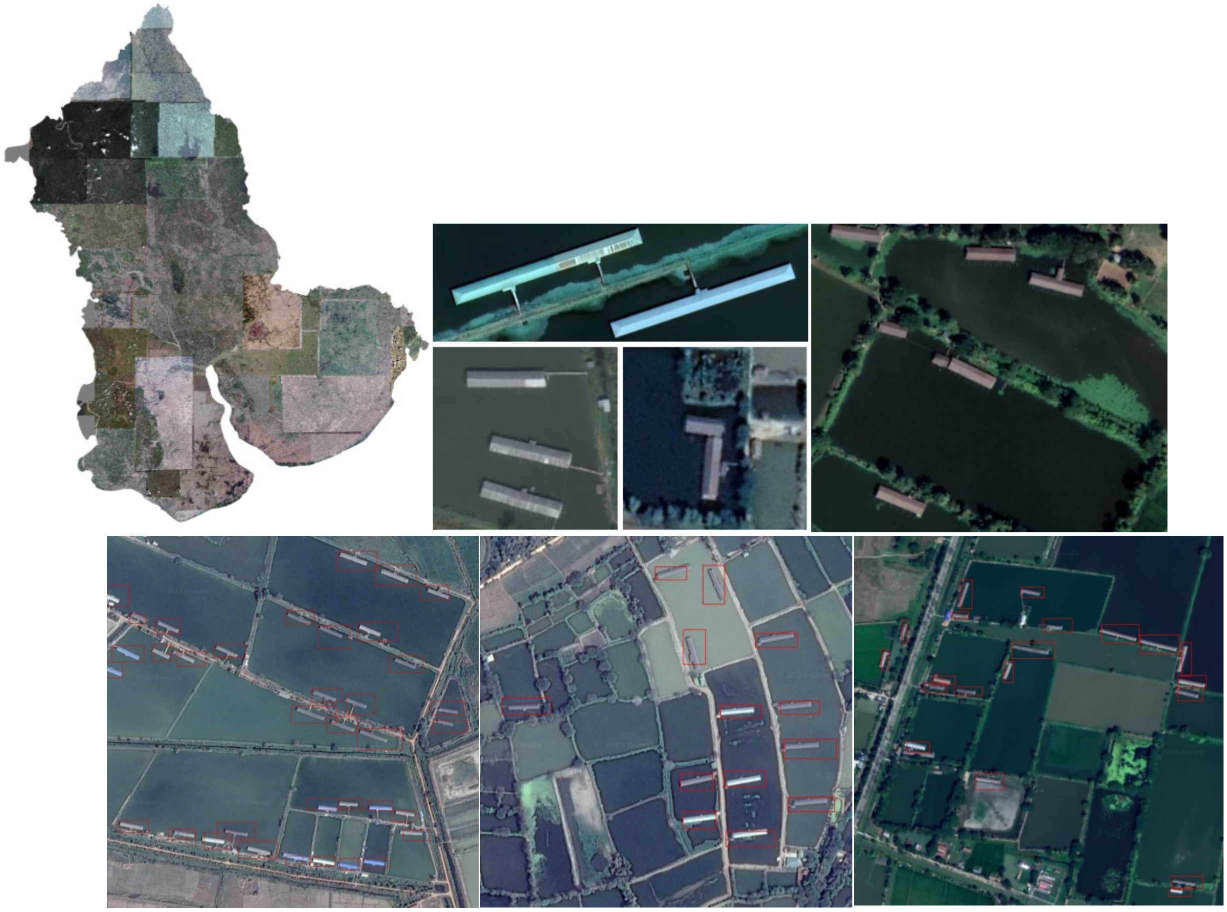

Supp. Fig. 1 | Sample satellite images of integrated chicken-fish farms

We used satellite images with a resolution of 1.19 meters per pixel for chicken house detection, and 0.59 meters per pixel for chicken house and fishpond segmentation. Downloading the higher

resolution map around detected chicken houses made it possible to reduce the computational burden without compromising segmentation precision.

We manually annotated a set of satellite images from Yangon region, taken in 2018. The annotations are in the form of bounding boxes, which can be used as ground-truth labels for training object detection models. As shown in Supp. Fig. 2, chicken houses are relatively obvious objects, but there is more fuzziness in human annotations of roofing type (thatch or zinc). We used survey data to verify the accuracy of our chicken house segmentation models.

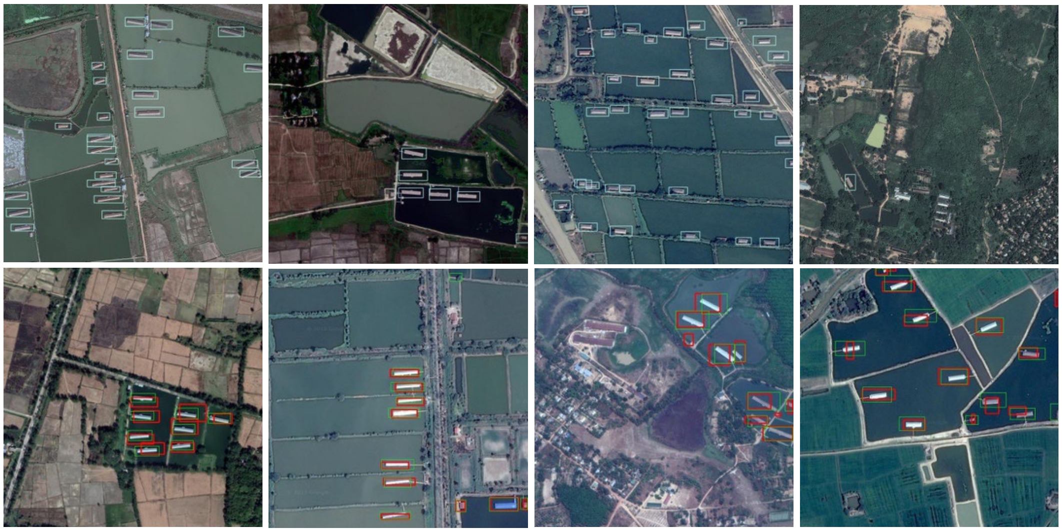

Supp. Fig. 2 | Visualization of the detection model

Note: The first row of the figures shows the predicted results for the year 2010. The bounding box predictions are marked in pale blue. The second row shows the predicted results on the test set of year 2018. The predictions are marked in green, and the annotation boxes are marked in red.

Chicken House Detection

To detect and count chicken houses, we employed object detection algorithms to locate and count individual chicken houses within satellite images. To analyze the characteristics of individual chicken houses, we employed a category-aware segmentation method. Segmentation algorithms were used to precisely delineate the boundaries of each chicken house, enabling us to accurately measure its size and assign a roof type (zinc or thatch).

YOLOv4[3] is a state-of-the-art object detection model known for its balance of speed and accuracy. We divided satellite images into manageable 608x608 pixel patches to accommodate the model's input requirements and enhance processing efficiency. To ensure seamless detection of houses spanning multiple patches, we strategically introduced overlaps between neighboring patches, guaranteeing that most houses would be fully captured in at least one patch. To achieve this, we set the overlap region width to be 96 pixels, which corresponds to approximately 114 meters, larger than the typical length of a chicken house. After cutting satellite images into fixed-sized patches, we applied the detection model to each individual patch. To effectively detect target farms located at the edge of different patches, we expanded each patch to overlap with its neighboring patches so that each chicken farm near the edge is completely included in at least

one patch. To prevent redundant detections caused by overlapping patches, we applied non-maximum suppression (NMS) during post-processing. This algorithm identifies and eliminates duplicate bounding boxes, resulting in accurate count estimates.

During both training and testing, we employed K-means clustering on a hold-out dataset to determine the optimal anchor box sizes specifically tailored to our task. Due to the special rotation- and flip-invariant property of satellite images, we also strengthened the data augmentation strategy of YOLOv4, including adding more rotation, flip, and mosaic operations. Rotations and flips simulate different image orientations, making the model invariant to object angles. Mosaic operations, which blend multiple images into a single composite, further enriched the training dataset with varied visual patterns.

To ensure the trustworthiness of our detection model, we employed a comprehensive suite of evaluation metrics: precision (P), recall (R), average precision (AP), and number accuracy (Acc). We used the Intersection-over-Union (IoU) metric for determining true positive detection results, calculated by the overlap between predicted bounding boxes and ground truthing boxes. Under IoU threshold k, Predictions with IoU above k are considered true positives. To evaluate model robustness, we tested it at different IoU thresholds, assessing its sensitivity to bounding box precision and ensuring reliable performance under diverse scenarios.

Supplementary Table 1 presents the model's precision, recall, average precision (AP), and number accuracy (Acc) under varying IoU thresholds (k equals 0, 0.2, and 0.5). Notably, the model achieves precision scores above 86% at IoU thresholds of 0 and 0.2. The model's ability to maintain a balanced error rate minimizes the risk of significant deviations in the overall count. In conclusion, while perfect detection is challenging in real-world data, the model's ability to achieve a balanced error rate and high number accuracy offers a reliable tool for estimating chicken house counts.

Supplementary Table 1 | Performance of the detection model

**IoU threshold (%)**	**Precision (%)**	**Recall (%)**	**Average Precision (%)**	**Accuracy (%)**
0	87.2	86.6	87.2	99.3
20	86.6	86.0	85.1	-
50	65.5	65.6	51.7	-

Supp. Figure 3 showcases the model's detection capabilities across diverse terrains. These images indicate that the model can reliably distinguish between chicken houses over ponds (i.e. integrated chicken-fish farms) and buildings of a similar appearance constructed on land. We hypothesize that generalization to other years is straightforward due to the consistent resolution and visual style of the satellite images. Future efforts to annotate chicken houses across various years will allow for a quantitative evaluation of our model's generalizability across time.

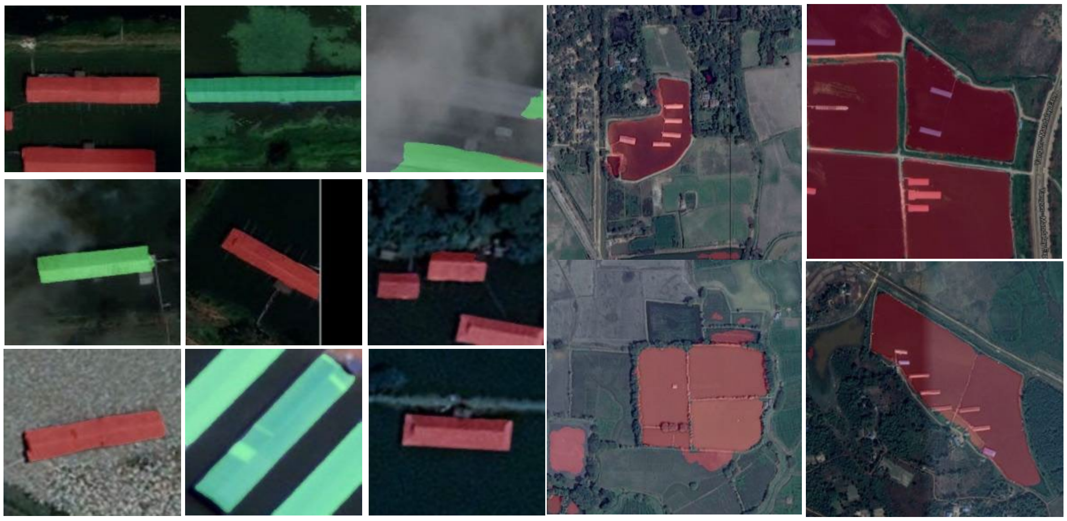

Supp. Fig. 3| Visualization of the U-net model on the validation set

Note: The bottom-left panel shows the segmentation and type classification of chicken farms. Zinc/metal farms are colored green and thatch farms are colored red. The bottom-right panel displays the segmentation of fishponds. The pond areas are colored red.

Chicken house segmentation and classification

To facilitate UNet[4] model training, we collected 569 satellite images of size 200*200px containing chicken houses estimated by our detection model. The image collection was annotated with the tight boundary of the chicken houses, along with the category of roofing material (zinc/thatch). For training, we split the labeled data into an 80% training set and a 20% validation set. The model predicts the category (zinc, thatch, or background) for each pixel in the input image. We use the cross-entropy loss between the estimated segmentation map and the ground truth segmentation mask as the loss function. For the architecture, we use ResNet34[5] as the backbone for the UNet model. Similar to the object detection model, we use a strong data augmentation strategy. Supplementary Table 2 presents the model's validation IoU.

Supplementary Table 2 | Performance of the segmentation model

Category of segmentation area	IoU (%)
Water/Fishpond	88.3
Chicken farm with thatched roof	83.0
Chicken farm with zinc roof	82.7

Accurate segmentation maps, visually demonstrating the model's ability to precisely delineate chicken house boundaries, are exhibited in Supp. Fig. 2. The model only fails in extreme cases,

such as heavy occlusion by clouds. For each segmented chicken house, we classified the roof material by comparing the per-pixel predictions. The material with the majority vote across the segmented area determines the classification (e.g., most zinc pixels classify the house as zinc, and similarly for thatch). Due to limitations in categorization, approximately 5% of roofs could not be definitively classified as either thatched or zinc-roofed. We evaluated the segmentation model's performance by applying it to our 2019 livestock field survey data and comparing its rooftop material classifications with the corresponding survey responses. The model achieved 68.5% accuracy, indicating good agreement with the survey data.

We reran the object detection using the most recent YOLO detection model, YOLOv11. The results from both models are very similar and YOLOv4 performs slightly better in some instances. We have therefore opted to retain the results based on YOLOv4 in the paper.

Primary surveys

We use supporting data obtained from two previous surveys of poultry farms; the Yangon Peri-urban Livestock Survey[6] (an in-person survey conducted in 2019), and the Yangon Peri-Urban Poultry Farmer Survey[7] (a six-round phone survey conducted in 2020).

During the Yangon Peri-urban Livestock Survey, in the absence of any publicly available registry of livestock farms, a sample frame was developed by analyzing satellite images to identify the location of poultry-fish farms, using the following steps. (1) Delimiting the geographical scope of the survey to an area within a 100 km radius of the center of Yangon city, determined using ArcGIS software. (2) Integrating shape files of village tract (sub-district) boundaries with Google Earth Pro software, to identify administrative units falling within 100 km of Yangon. (3) Conducting a systematic visual search of satellite images of this zone, village tract by village tract, in Google Earth Pro, to identify integrated chicken-fish farms. The location of all integrated chicken-fish ponds identified was recorded in a geo-coded database and all chicken houses visible over ponds were counted and logged. (4) Estimating the density per km^2 of integrated chicken-fish houses in each village tract in the zone using the count of chicken houses and ranking the number and density of integrated farms per village tract to identify village tracts with high concentrations for inclusion in the sample frame. (5) Validating village tract selection through consultation with key informants and combining information from the visual search and key informants to select 83 village tracts with high concentrations of farms. (6) Selecting villages for inclusion in the survey from selected village tracts by probability proportional to population size.

A complete listing of livestock farms was conducted in all selected villages. The minimum size for inclusion of poultry farms in the survey was set at 500 chickens per farm, as the survey was designed to capture information on commercial farming operations. All farms in selected villages at or above this threshold were selected with 100% probability. The survey questionnaire was designed to collect detailed information on farm production practices and production economics. Interviews were conducted face to face with farm owners by trained enumerators on the respondents' farms. The final dataset is comprised of data from 513 farms, of which 423 chicken farms.

Rounds one to four of the Yangon Peri-Urban Poultry Farmer Survey were fielded biweekly in June and July 2020. Rounds five and six were conducted in August and November, respectively. The survey was based on a subsample of 269 chicken farms (190 broiler and 79 layer) included in the 2019 Yangon Peri-urban Livestock Survey. The subsample comprised all the broiler and layer farms from the original sample that could be contacted by phone and agreed to be interviewed. 74% of surveyed broiler farms and 52% of layer farms were integrated with fishponds. The survey questionnaire collected information on farm operational status, business operations, and business sentiment.

Validating the reliability of satellite images as a data source

Our analysis assumes that farms that close are more likely to remove chicken houses than those remaining operational. We verified this assumption by examining satellite images for a subsample of 85 farms included in our 2020 phone survey for which geolocated data on ponds were available. We checked whether the closure of farms, as reported in the phone survey, was associated with the disappearance of chicken houses from satellite images in 2020 and 2021. Our visual assessment of satellite images indicated that 9% of farms that were operational throughout 2020 experienced a reduction in the number of chicken houses by 2021. In contrast, 60% of farms that closed in 2020 were found to have lost chicken houses by 2021 (Supplementary Table 3). This observation confirms that there is a strong association between non-operational farm status and removal of chicken houses. The exercise also suggests that our estimates of declines in chicken and egg supply due to farms halting production or closing are conservative, given that not all non-operational farms removed chicken houses immediately.

Supplementary Table 3 | Share of the integrated chicken-fish farms with a decreasing number of chicken houses since early 2020 based on 2020/21 satellite images, by operational status

Operation status	Permanently closed farms	Operational farms
% of farms with fewer chicken houses	60%	9%
Sample N	*10*	*75*

Source: Google Earth satellite images 2020-21; Yangon peri-urban livestock survey 2019.

Note: We conducted a visual search of Google Earth historical satellite images to determine if the number of chicken houses in the integrated fish-chicken farms had decreased in 2020/21 (i.e., disappeared from the 2020/21 satellite images). The sample size here is smaller than the phone survey because we only looked at farms with collected GPS data.

While satellite images allow us to detect integrated farms with a high degree of accuracy, distinguishing between integrated chicken-fish and pig-fish farms remains a challenge as they look similar on satellite images. In Supplementary Table 4, we assume that 96% of integrated houses are chicken-fish farms, and 4% are pig-fish farms, based on the composition of farms in the Yangon Peri-urban Livestock Survey 2019. Additionally, we adjusted the area of chicken houses because the area detected from satellite images is the roof, which is slightly larger than the floor. We estimated the ratio of floor area to roof area at 0.95, based on the 2019 field survey and converted roof area to chicken house area using this ratio.

Supplementary Table 4 | Number and size of integrated chicken-fish houses by roof type

Year	Total number of thatched houses	Total number of zinc-roofed houses	Total area of thatched houses (m^2)	Total area of zinc-roofed houses (m^2)	Average size of thatched houses (m^2)	Average size of zinc-roofed houses (m^2)
2010	804	130	331,129	49,070	412	379
2011	865	121	360,090	51,208	416	423
2012	914	201	383,433	91,685	420	457
2013	938	229	394,941	104,817	421	457
2014	916	443	408,112	237,684	446	537
2015	844	553	377,549	287,399	447	520
2016	856	640	364,913	353,100	426	551
2017	908	763	424,130	407,335	467	534
2018	989	1,243	441,297	670,588	446	539
2019	1,082	1,313	494,700	725,780	457	553
2020	1,156	1,253	527,929	686,084	457	548
2021	1,088	969	508,338	543,052	467	561
2023	1,096	763	539,707	469,786	492	616
Overall change 2010-20 (%)	43.8	866.7	59.4	1,298.2	10.8	44.6
Overall change 2020-23 (%)	-5.1	-39.1	2.2	-31.5	7.8	12.4
Annual change 2010-20 (%)	2.6	30.3	3.8	34.8	1.1	3.5
Annual change 2020-23 (%)	-1.4	-14.7	1.1	-11.2	2.5	4.1

Source: Google Earth satellite images 2010-23.

To estimate change in the standing population of broiler and layer chickens we decomposed total farm area into the area devoted to broilers and layers, based on the results of the 2019 field survey. We then multiplied the area of each type of integrated chicken-fish house in 2020 and 2023 by the density of each type of chicken to obtain the estimated standing population of chickens in each year. We subsequently converted these figures to annual chicken and egg supply using the average number of broiler production cycles per year and average number of eggs laid per layer reported in the 2019 field survey (Supplementary Table 5).

Supplementary Table 5 | Estimated annual supply of broilers and layers from integrated chicken-fish farms in Yangon Region, 2020 and 2023

	Broiler	Layer
Density of chickens raised on integrated farms (birds/m²)[1]		
A: Chicken density	5.7	10.9
Area of chicken houses (million m²)[3]		
B1: 2020	1.0	0.3
B2: 2023	0.8	0.2
Standing population of chickens (million)		
C1: 2020 (=A*B1)	5.7	3.2
C2: 2023 (=A*B2)	4.7	2.6
C3: Change, 2020-2023 (=C2-C1)	-1.0	-0.55
Annual supply of broilers (birds) and eggs (million)		
D1: Broiler production cycles / eggs laid per layer, per year[1]	5	206
D2: Supply in 2020 (=D1*C1)	28.6	651.6
D3: Supply in 2023 (=D1*C2)	23.6	537.7
D4: Change, 2020-2023 (=D3-D2)	-5.0	-113.9
Estimated change in annual consumption of chickens and eggs in Yangon		
E0: Average weight (kg) per live chicken[1]	2.7	
E1: Average weight (kg) per dressed chicken[2] and per egg[1]	1.9	0.05
E2: Change in annual supply of broilers and eggs (million kg), 2020-2023 (=D*E1)	-9.3	-5.7
E3: Human population of Yangon in 2020 (in million)	7.9	7.9
E4: Change in annual consumption (kg/per capita) of broilers and eggs, 2020 to 2023 (=E2/E3)	-1.2	-0.7
E5: Change in annual consumption of individual birds and eggs, 2020 to 2023 (=E4*E1)	-0.6	-14
Change in annual consumption of chicken and eggs relative to consumption in 2015[4]		
F1: Consumption of chicken and eggs in Yangon in 2015 (kg/capita/year)	11.0	4.4
F2: Change in annual consumption of chicken and eggs from integrated farms in Yangon in 2023, relative to 2015 (=E4/F1)	-10.7%	-16.4%

Source:
[1]Yangon peri-urban livestock survey 2019.
[2]USDA FoodData Central[8] (edible portion of a whole chicken is 69%)
[3]Google Earth satellite images
[4]Myanmar Poverty and Living Conditions Survey 2015

To evaluate the impacts of the change in chicken and egg supply on human consumption of these products we calculated the change in the total number of birds and eggs produced in 2020 and 2023 and multiplied these values by the average weight of individual chickens and eggs reported in the 2019 field survey. These values were divided by the human population of Yangon region

in 2019 reported by the Myanmar Department of Population Yangon population[9], and adjusted upward using the ratio of national population estimates for 2019 and 2020 reported by FAOSTAT[10].

Finally, we used data extracted from the dataset of the Myanmar Poverty and Living Conditions Survey 2015[11], which is the most recent publicly available representative household survey containing high-quality data on food consumption, to evaluate chicken and egg consumption in Yangon region.

Supplementary References

1. Google Earth. Google Earth Historical imagery of Yangon Province 2010-2023. Preprint at (2022).
2. (LBVD) Livestock Breeding and Veterinary Department. *Report of National Livestock Baseline Survey 2018*. (2019).
3. Bochkovskiy, A., Wang, C.-Y. & Liao, H.-Y. M. YOLOv4: Optimal Speed and Accuracy of Object Detection. (2020).
4. Ronneberger, O., Fischer, P. & Brox, T. U-net: Convolutional networks for biomedical image segmentation. *Lecture Notes in Computer Science (including subseries Lecture Notes in Artificial Intelligence and Lecture Notes in Bioinformatics)* **9351**, 234–241 (2015).
5. He, K., Zhang, X., Ren, S., recognition, J. S. pattern & 2016, undefined. Deep residual learning for image recognition. in *openaccess.thecvf.com*.
6. Belton, B., Cho, A., Payongayong, E., Mahrt, K. & Abaidoo, E. *Commercial Poultry and Pig Farming in Yangon's Peri-Urban Zone*. https://ageconsearch.umn.edu/record/303953/ (2020).
7. Fang, P., Belton, B., Zhang, X. & Ei Win, H. Impacts of COVID-19 on Myanmar's chicken and egg sector, with implications for the sustainable development goals. *Agric Syst* **190**, 103094 (2021).
8. USDA (US Department of Agriculture). Agricultural Research Service. FoodData Central. fdc.nal.usda.gov. (2019).
9. Myanmar Department of Population. Myanmar Population estimate 2019. https://dop.gov.mm/en/publication-category/main-report-0 (2020).
10. Food and Agriculture Organization (FAO). FAOSTAT Annual population. https://www.fao.org/faostat/en/#data/OA (2024).
11. Scott, J. M., Belton, B., Mahrt, K., Thilsted, S. H. & Bogard, J. R. Food systems transformation, animal-source foods consumption, inequality, and nutrition in Myanmar. *Food Secur* **15**, 1345–1364 (2023).
