# Supplementary material for: Geospatial analysis enables combined poultry–fish farm monitoring in the fragile state of Myanmar
Source: Nat Food. 2025 Jul 23;6(7):664–7. doi: 10.1038/s43016-025-01192-1 (PMC12454148; doi:10.1038/s43016-025-01192-1)
Supplement: Supplementary file 2 — Reporting Summary [file 43016_2025_1192_MOESM2_ESM.pdf]

nature portfolio

Corresponding author(s): Xiaobo Zhang

Last updated by author(s): Feb 21, 2025

Reporting Summary

Nature Portfolio wishes to improve the reproducibility of the work that we publish. This form provides structure for consistency and transparency in reporting. For further information on Nature Portfolio policies, see our Editorial Policies and the Editorial Policy Checklist.

Statistics

For all statistical analyses, confirm that the following items are present in the figure legend, table legend, main text, or Methods section.

n/a	Confirmed	
☐	☒	The exact sample size (*n*) for each experimental group/condition, given as a discrete number and unit of measurement
☐	☒	A statement on whether measurements were taken from distinct samples or whether the same sample was measured repeatedly
☐	☒	The statistical test(s) used AND whether they are one- or two-sided<br>*Only common tests should be described solely by name; describe more complex techniques in the Methods section.*
☐	☒	A description of all covariates tested
☐	☒	A description of any assumptions or corrections, such as tests of normality and adjustment for multiple comparisons
☐	☒	A full description of the statistical parameters including central tendency (e.g. means) or other basic estimates (e.g. regression coefficient) AND variation (e.g. standard deviation) or associated estimates of uncertainty (e.g. confidence intervals)
☒	☐	For null hypothesis testing, the test statistic (e.g. *F*, *t*, *r*) with confidence intervals, effect sizes, degrees of freedom and *P* value noted<br>*Give P values as exact values whenever suitable.*
☒	☐	For Bayesian analysis, information on the choice of priors and Markov chain Monte Carlo settings
☐	☒	For hierarchical and complex designs, identification of the appropriate level for tests and full reporting of outcomes
☒	☐	Estimates of effect sizes (e.g. Cohen's *d*, Pearson's *r*), indicating how they were calculated

Our web collection on statistics for biologists contains articles on many of the points above.

Software and code

Policy information about availability of computer code

Data collection	1) Survey Solutions (https://mysurvey.solutions/en/) was used to collect the the Yangon Peri-urban Livestock Survey data 2019, and Google Earth Pro and ArcGIS were used to develop the survey sample frame. 2) SurveyCTO was used to collect the Yangon Peri-Urban Poultry Farmer Survey data 2020. 3) Google Earth Pro was used to access the satellite images for chicken house detection.
Data analysis	1) StataSE Version 17 was used to analyze the two sets of survey data (do files are published on Github). 2) Python was used for the chicken-fish house detection, roof material grouping, and roof area estimate (codes are published on Github).

For manuscripts utilizing custom algorithms or software that are central to the research but not yet described in published literature, software must be made available to editors and reviewers. We strongly encourage code deposition in a community repository (e.g. GitHub). See the Nature Portfolio guidelines for submitting code & software for further information.

Data

Policy information about availability of data

All manuscripts must include a data availability statement. This statement should provide the following information, where applicable:

- Accession codes, unique identifiers, or web links for publicly available datasets
- A description of any restrictions on data availability
- For clinical datasets or third party data, please ensure that the statement adheres to our policy

The Yangon Peri-urban Livestock Survey 2019 dataset is archived on Harvard Dataverse at: https://dataverse.harvard.edu/dataset.xhtml?persistentId=doi%

3A10.7910%2FDVN%2FLKAQYF&version=DRAFT

Yangon Peri-Urban Poultry Farmer Survey 2020 dataset is archived on Harvard Dataverse at: https://dataverse.harvard.edu/dataset.xhtml?persistentId=doi%3A10.7910%2FDVN%2FIY66P9&version=DRAFT
All STATA code used in the analysis of primary survey data and estimates of poultry and egg supply is available at: https://github.com/Percyfang/Yangon-Chicken-Fish.
All machine learning code used in chicken house detection, segmentation and classification is available as: https://github.com/PKUCER/YONGONCHICKENFISH

Human research participants

Policy information about studies involving human research participants and Sex and Gender in Research.

Reporting on sex and gender	Gender of business owners was collected in both farmer surveys, but this information s not a variable of interest in this study and is not used in our analysis
Population characteristics	The respondents to the two surveys are the owners of integrated commercial livestock farms within a 100 km radius of Yangon in Yangon region, Ayeyarwady region, and Bago region of Myanmar. The average age of farm owners was 44. 26% of farm owners were women
Recruitment	We deployed several strategies to obtain information on locations and numbers of integrated poultry-fish farms in the Yangon peri-urban zone, from which to develop a sample frame. First, we identified locations with integrated poultry-fish farms by analyzing satellite images, by the following steps. (1) We delimited the geographical scope of the survey to the area within a 100 km radius of the center of Yangon city, and determined the extent of this zone using ArcGIS software. (2) We integrated shape files of township (district) and village tract (sub-district) boundaries into Google Earth Pro allowing us to identify the all the administrative units falling within 100 km of Yangon. (3) We conducted a systematic visual search of satellite images of this zone, village tract by village tract, in Google Earth Pro, to identify integrated chicken-fish farms. Wherever integrated chicken-fish ponds were identified, the location was recorded in a geo-coded database and the number of chicken houses was counted and logged. Results from the initial sweep of satellite images were rechecked and validated visually. This information allowed us to count the number and calculate the density (per km2) of integrated chicken-fish houses per village tract within the zone of interest. Ranking village tracts by number and density of farms made it possible to identify village tracts with high concentrations of integrated farms for inclusion in the sample frame. We selected a pool of 83 village tracts with the highest concentrations of chicken farms. Villages were selected for survey from each village tract randomly, by probability proportional to size. A complete listing of pig and poultry farms was conducted in all selected villages. The listing included information on type of animal raised and flock or herd size. The survey was designed to capture information on strongly commercially oriented farming operations, so the minimum size threshold for inclusion in the survey was set at 500 or more chickens. All farms in selected villages that met these size criteria were selected for inclusion in the survey with 100% probability. This process generated a total dataset of 423 chicken farms.
Ethics oversight	The Yangon Peri-urban Livestock Survey 2019 was determined exempt under Michigan State University IRB# x13-635e.<br>The Yangon Peri-Urban Poultry Farmer Survey 2020 received IRB approval from the International Food Policy Research Institute for IRB #00007490

Note that full information on the approval of the study protocol must also be provided in the manuscript.

Field-specific reporting

Please select the one below that is the best fit for your research. If you are not sure, read the appropriate sections before making your selection.

☐ Life sciences ☒ Behavioural & social sciences ☐ Ecological, evolutionary & environmental sciences

For a reference copy of the document with all sections, see nature.com/documents/nr-reporting-summary-flat.pdf

Behavioural & social sciences study design

All studies must disclose on these points even when the disclosure is negative.

Study description	Data from the two poultry farm studies are quantitative data derived from structured surveys: the first one was conducted in-person and the second one was phone survey. All the responses were self-reported by survey respondents. The data extracted from satellite image detection is geo-spatial, and estimates the number of integrated chicken-fish farms, further categorized by type of roofing material, and the area of chicken houses.
Research sample	Respondents to the Yangon Peri-urban Livestock Survey data 2019 survey are the owners of integrated commercial livestock farms within a 100 km radius of Yangon in Yangon region, Ayeyarwady region, and Bago region of Myanmar. This is the most important chicken production area in Myanmar and serves the largest city: Yangon city. The survey was designed to capture information on commercially oriented farming operations, so the minimum size threshold for inclusion in the survey was set at 500 or more chickens. All farms in selected villages that met these size criteria were selected for inclusion in the survey with 100% probability. The sample should therefore be considered representative only of the farms selected. Nevertheless, the fairly uniform nature of production practices reported among most types of farm in our sample means it is likely that the results are comparable to those for

	other farms in the same size range within the zone surveyed.<br>The respondents of the 2020 Yangon Peri-Urban Poultry Farmer Survey were a subset of of participants from the 2019 survey. We included all those broiler or layer farmers who could be successfully contacted using the phone numbers collected in the 2019 survey, resulting in a sample of 269 chicken farmers.<br>Our study using satellite imagery is focused exclusively on Yangon Region, an administrative region that includes Yangon City within its boundaries. Therefore satellite images were selected to cover the entire area of Yangon Region, Myanmar.
Sampling strategy	As reported above, we deployed a mix of strategies to obtain information on locations and numbers of integrated poultry-fish farms in the Yangon peri-urban zone, from which to develop a survey sample frame for the 2019 Yangon Peri-urban Livestock Survey. We selected a pool of 83 village tracts within a 100 km radius if Yangon city with the highest concentrations of chicken farms. Villages were selected for survey from each village tract randomly, by probability proportional to size. A census of poultry farms was conducted in all selected villages. This list included information on type of animal raised and flock or herd size. The minimum size threshold for inclusion in the survey was set at farms with a standing population of 500 or more chickens. All farms in selected villages that met these size criteria were selected for inclusion in the survey with 100% probability. This process generated a total dataset of 423 chicken farms. The Yangon Peri-Urban Poultry Farmer Survey data 2020 resurveyed a sub-sample of 269 chicken farms included in the 2019 survey. This was the complete set of farms that could be contacted again by phone and agreed to participate in the survey.
Data collection	The 2019 Yangon Peri-urban Livestock Survey was programmed in Survey Solution, and the 2020 Yangon Peri-Urban Poultry Farmer Survey was programmed in SurveyCTO. Local supervisors in Myanmar trained enumerators to use these platforms on tablets to conduct both surveys. The 2019 survey involved face-to-face interviews between an enumerator and a business owner or manager, whereas the 2020 survey, due to COVID-19, was conducted one-on-one by phone. For both surveys, the interview data were typically uploaded on the same day the interview was completed.
Timing	The Yangon Peri-urban Livestock Survey 2019 was conducted between mid-August and mid-September 2019. The Yangon Peri-Urban Poultry Farmer Survey 2020 was conducted over six rounds between May to November 2020.
Data exclusions	No data were excluded from the analysis.
Non-participation	Only 1 out of 514 respondents rejected the interview during the Yangon Peri-urban Livestock Survey 2019. For the Yangon Peri-Urban Poultry Farmer Survey 2020, 17 out of 269 farms completely closed during June and November survey rounds, so they were dropped.
Randomization	Participants were not allocated into experimental groups.

Reporting for specific materials, systems and methods

We require information from authors about some types of materials, experimental systems and methods used in many studies. Here, indicate whether each material, system or method listed is relevant to your study. If you are not sure if a list item applies to your research, read the appropriate section before selecting a response.

Materials & experimental systems

n/a	Involved in the study	
☒	☐	Antibodies
☒	☐	Eukaryotic cell lines
☒	☐	Palaeontology and archaeology
☒	☐	Animals and other organisms
☒	☐	Clinical data
☒	☐	Dual use research of concern

Methods

n/a	Involved in the study	
☒	☐	ChIP-seq
☒	☐	Flow cytometry
☒	☐	MRI-based neuroimaging
